# Supplementary material for: Are carbon emissions trading and green financial instruments synergistic? -Comprehensive quantitative research based on content analysis
Source: PLoS One. 2024 Mar 7;19(3):e0298601. doi: 10.1371/journal.pone.0298601 (PMC10919601; doi:10.1371/journal.pone.0298601)
Supplement: S1 Data — (ZIP) [file pone.0298601.s001.zip › Data/地级市Z_F工作报告.zip!/╜¡╬≈/╕o╓▌╩╨/└┤╫╘╣1⁄2╓┌║┼í╛┬φ┐╦╩2╛▌═°í┐_2017.docx]

各位代表：

　　现在，我代表市人民政府向大会报告工作，请予审议，并请各位市政协委员和列席会议的同志提出意见。

　　一、真抓实干，经济社会发展迈出坚实步伐

　　市第三届人民代表大会第一次会议以来的五年，是抚州发展史上极不平凡的五年，是经济社会大发展、综合实力大提升、城乡面貌大变化、群众生活大提质的五年。五年来，特别是市委三届十一次全会以来，在省委、省政府和市委的正确领导下，在市人大、市政协的监督和支持下，市人民政府主动适应经济发展新常态，全力应对“十二五”中后期经济下行压力，以新发展理念为引领，全力打好“三大攻坚战”，凝心聚力，攻坚克难，顺利完成了市三届人大各次会议确定的主要任务，为全面建成小康新抚州奠定了坚实基础。

　　（一）综合实力进一步增强，绿色发展实现新突破。

　　1.经济发展提速进位。五年来，全市生产总值上升到1105.14亿元，年均增长10.5%；财政总收入165.2亿元，年均增长17.4%；公共财政预算收入126.82亿元，年均增长18.37%；500万元以上固定资产投资1099.67亿元，年均增长18%；社会消费品零售总额428.2亿元，年均增长13.2%；实际利用外资3.86亿美元，年均增长13.7%；出口总额16.5亿美元，年均增长26.82%。其中，财政总收入、公共财政预算收入实现四年翻番；生产总值和财政总收入在全省排名双双前移；财政收入超10亿元县（区）由1个增至8个，东乡、临川突破20亿元。三次产业比调整为2015年的16.5：49.7：33.8。特别是2015年，生产总值、500万元以上固定资产投资双双突破千亿元大关。

　　2.工业经济壮大升级。五年来，规模以上工业增加值上升到364.2亿元，年均增长13.8%。主营业务收入过百亿园区从1个增至6个。2015年抚州高新区成功晋级国家级高新技术产业开发区。产业集群发展态势良好，新增省级产业基地6个，总量居全省前列；六大主导产业主营业务收入和利税总额分别增长180%和302%，六大主导产业占工业主营业务收入比重达到67.5％。新增规模以上企业325户，规上企业总数达到903户。特别是2015年，规模以上工业增加值和工业企业利税总额在全省排名各前进2位。工业税收突破40亿元大关，成为第一大税源。

　　3.现代农业提质增效。粮食生产实现“十二连增”，产量达293.1万吨，较2010年增长11.5%。农业总产值增长57.5％，形成了十大农业产业集群。全市共有无公害农产品290个，绿色食品29个，有机产品67个，获得中国驰名商标5个，农产品地理标志登记保护认证产品21个。农民人均纯收入达到11441元，增幅连续5年高于城镇居民收入和GDP增速。特别是2015年，临川区被列为国家级现代农业示范园区，7个县被列为省级现代农业示范园区。苍源药业成为全省第一家在“新三板”挂牌的农业企业。全面完成480万亩耕地保护和149万亩高标准农田建设任务。

　　4.现代服务业快速发展。2015年，服务业增加值占GDP比重提高1.4个百分点，对经济增长的贡献率近40%。休闲旅游、文化创意、中医药、健康养老、现代物流、信息经济等六大新兴产业扎实推进。全市有1家企业在主板上市，7家企业在“新三板”挂牌，38家企业在江西股权交易中心挂牌。抚州海西综合物流园、抚州农产品物流中心等一批物流基础设施项目开工建设。特别是2015年以来，资溪大觉山景区入选国家5A级景区预备名单。资溪、南丰被列为首批全域旅游示范区创建单位。文化产业主营业务收入达到236.58亿元，实现五年翻两番。

　　（二）人居环境进一步优化，城乡建设呈现新面貌。

　　1.重大项目快速推进。五年来，累计完成基础设施建设投资833.1亿元，年均增长23.3%。抚州人民的铁路梦、电厂梦、高速梦、汽车梦“新临川四梦”相继实现。向莆铁路、沪昆客专、抚吉高速、昌宁高速、抚金高速、抚州电厂一期工程、500千伏抚州—红都输电线路工程、西气东输二线工程、省天然气管网一期工程抚州段等一批重大基础设施工程先后竣工。特别是2015年以来，一批事关抚州长远发展的重大项目取得突破。东外环高速征地基本完成，11月上旬开工建设。昌抚城际铁路正在加快前期工作。鹰梅铁路已纳入国家中长期铁路网调整规划，吉抚武铁路“十三五”内将动工建设。抚州机场建设已进入实质性启动阶段。廖坊水利枢纽灌区二期工程正式开工建设。

　　2.城镇化建设进程加快。五年来，实施重点城建项目172个，累计完成投资218亿元。市中心城区建成区面积达到63平方公里，扩大12.7平方公里。全市城镇化率达45%，年均提高1.39个百分点。市中心城区人均公园绿地面积、绿地率和绿化覆盖率均居全省前列。成功创建“国家园林城市”、“国家森林城市”、“省级文明城市”和“省级卫生城市”。先后开通抚州至东乡、抚州至金溪城际公交。特别是2015年以来，强力推进“五车”整治、高速公路沿线广告整治、交通运输超载超限整治、河道违法采砂专项整治，城市形象、市民文明程度进一步提升。

　　3.新农村建设成效明显。五年来，共投入资金26.4亿元，完成4435个新农村点建设，惠及18.61万户、74.7万人，改水、改厕到位率均达95%以上，改路率达100%。在37个乡镇实施了镇村联动建设，累计投入资金5亿元，辐射带动周边村庄465个，打造了一批镇村融合、城乡统筹的示范镇，初步形成了以镇带村、以村促镇的发展新格局。

　　4.生态优势日益凸显。节能减排成效显著，单位生产总值能耗比“十一五”下降13%。黎川、南丰、宜黄、资溪、广昌5县纳入国家重点生态功能区。抚河源纳入国家生态补偿试点范围，抚河水质达标率100%，集中式饮用水水源地水质达标率100%。在抚河干流沿岸规划建设36个生态村镇示范点，创建国家级生态乡镇16个。市、县、乡、村四级“河长制”、“山长制”全面建立。特别是2015年，在全省率先启动生态文明先行示范市建设，率先在全省实施全域性封山育林。在“中国大陆城市‘氧吧’50强”中位列第一。总投资370亿元的抚河流域生态保护及综合治理工程启动实施。

　　（三）发展活力进一步激发，改革开放迈出新步伐。

　　1.重点改革深入推进。重要领域和关键环节改革取得明显进展。启动改革试点、示范及试验区建设56个，公务消费一体化监管改革、农村“三资”监管改革、农村综合产权制度改革、教育综合改革等试点走在全省前列。启动南城省管县改革和东乡撤县设区工作。实施临川教育集团转型升级和市中心城区义务教育就近免试入学。党政机关公务用车改革全面完成，乡镇综合行政执法改革和金溪、南城县的乡镇便民服务中心建设试点顺利开展。商事制度改革稳步推进，社会信用体系建设步伐加快。

　　2.区域合作全面深化。深化区域合作加快发展上升为省级战略。四个重点平台列入省原中央苏区振兴重点平台建设。与闽浙赣、闽粤赣、闽浙赣皖区域合作组织联系对接进一步加强。特别是2015年以来，昌抚合作发展深入推进，《昌抚合作示范区规划》获省发改委批准，昌抚城际铁路列入省“十三五”重大项目。《赣闽台产业合作示范区规划》、《江西向莆经济带“十三五”发展规划》获省政府批复，已全面启动规划建设。

　　3.对外开放成效显著。五年来，新引进并已开工亿元以上项目738个，其中5亿元以上项目192个，竣工投产540个。投资50亿元的海利不锈钢、35亿元的天津卓朗、30亿元的森鸿科技、20亿元的迪比科新能源、15亿元的国药中药饮片、13.5亿元的博雅欣和等一批重大产业项目落地建设。企业“走出去”步伐加快。新增6家对外投资企业，新增5家对外承包工程资质企业。中阳集团积极开拓赞比亚、吉布提等非洲国家市场，已达成多个工程建设项目合作。

　　4.创新能力不断提升。全市科技进步贡献率达50.1%，比“十一五”提高5.3个百分点。高新技术产业增加值年均增长21.6%。新增国家高新技术开发区1个、国家级高新技术产业化基地1个、省级高新技术产业化基地3个、省部共建国家重点实验室1个、国家级工程研究中心1个。先后与180多家高校、科研机构开展合作，合作项目达207个。国家科技富民强县工程项目实现县（区）全覆盖，被评为全国科技进步先进市。“双返双创”扎实推进，以博雅欣和、迪比科、德义半导体为代表的创新型企业快速壮大，大众创业、万众创新热潮涌动。

　　（四）人民生活进一步改善，社会建设取得新成就。

　　1.脱贫攻坚成效显著。坚持精准识别，综合运用产业扶贫、安居扶贫、保障扶贫、健康扶贫、教育扶贫等多种方式加大脱贫攻坚力度。五年来，全市共完成253个贫困村和扶贫对象建档立卡工作，完成移民搬迁3.4万余人，贫困人口由31.6万人下降到12.6万人，全市城乡低保对象的补助标准全面提高。重点实施产业扶贫，通过加快产业发展从根本上提升贫困地区脱贫致富能力。截至2015年底，全市产业扶贫项目已覆盖90%以上贫困村，参与产业开发贫困群众人均增收800元以上。

　　2.民生状况持续改善。五年来，全市民生支出747.4亿元，占公共财政预算支出比重达到63.7%。新增城镇就业24.9万人，新增农村劳动力转移就业33.8万人，城镇登记失业率控制在3.3%以内。2015年城镇居民人均可支配收入25065元，是2010年的1.73倍。城乡基本医疗保险、大病保险、重特大疾病医疗保障和医疗救助四条保障线全面建立。五年完成城镇保障性安居房45083套，完成棚户区改造21161户；新建和改造农村危旧房57239户。特别是2015年以来，全面启动全省规模最大、历史遗存最完整的文昌里棚户区改造和历史文化街区建设项目。市中心城区启动11个重点棚改项目，市县棚改征迁面积达到160万m2。

　　3.社会事业长足进步。基础教育走在全省前列。实行名校带民校，通过PPP模式竞争引进社会资本12亿元新建3所民办学校，增加优质学位2.4万个。投资近24亿元建设市妇女儿童医院、市第一医院改造等8个卫生项目，实施并基本完成每个行政村建设1所标准化卫生计生服务室。新增国家一级图书馆1个、国家一级文化馆1个。成功举办纪念汤显祖逝世400周年暨中国（抚州）汤显祖艺术节系列活动，乡音版（旴河高腔）《临川四梦》在国家大剧院、清华、北大成功上演。启动汤显祖作品进教材和多语种翻译工程。汤显祖作为中华文化的标志性符号，已深深镌刻在全球华人心中。临川文化正在充满自信地大踏步走向世界舞台。国防动员和后备力量建设持续推进，成功组织“红土地2015”国防动员演习，国防教育、民兵基层、兵员征集、“双拥”共建等工作取得新的成效。体育、卫生和人口计划生育、食品药品监管、国资监管、市场与质量监管、行政司法、民族宗教、外事侨务、涉台事务、审计、粮食、统计调查、人民防空、防震减灾、气象、水文、档案、地方志、妇女儿童、老龄、残联、科协、红十字会、驻外联络等工作有了新的进步。

　　4.社会秩序和谐稳定。积极化解信访矛盾纠纷，引导群众理性合法表达诉求，信访总量、重复信访量、越级信访量和集体上访量均呈下降趋势。社会管理“网格化”、重大事项风险评估、领导干部“一岗双责”、“第三方”化解信访矛盾等工作机制，多次受到中央和省里的充分肯定。积极开展以打击“盗、抢、骗”违法犯罪为主的“飓风”行动，始终保持对各类犯罪活动的高压严打态势，有力保障了社会和谐稳定。安全生产状况明显好转，安全生产事故比“十一五”下降60.29％。

　　（五）行政效能进一步提升，自身建设展示新形象。

　　1.“四风”问题整治扎实有效。认真开展党的群众路线教育实践活动、“三严三实”专题教育、“两学一做”学习教育，针对“四风”突出问题，落实了整改措施，完善了长效机制。建立健全政策落实的责任机制、奖勤罚懒的激励机制和常态化的督查问责机制。

　　2.行政权力运行规范有序。建立网上审批和电子监察系统，推行风险岗位廉能管理，强化经济责任审计和重点项目审计，有力推动了政府系统党风廉政建设和反腐败工作。严格落实党风廉政建设主体责任制，坚持“一岗双责”。

　　3.法治政府建设步伐加快。顺利完成“六五普法”，民主法制建设全面推进。建立法治政府建设指标体系，坚持把法治政府建设成效作为衡量各级领导班子和领导干部工作实绩重要内容。全面推行政府法律顾问制度、落实行政机关负责人出庭应诉制度。自觉接受市人大及其常委会的依法监督、工作监督和市政协的民主监督。累计办理市人大代表建议738件，市政协提案1312件，满意率和基本满意率均达98%以上。

　　各位代表，五年的奋斗历程，给予我们许多深刻的启示，也为今后发展积累了宝贵经验：

　　一是必须坚持走绿色发展道路。绿色发展不是不要发展，也不等于慢发展，而是换一种思维抓发展，关键是发展新经济，培育新动能，促进经济转型升级。实践证明，绿色生态是抚州最大的财富、最大的品牌、最大的优势。进入生态文明新时代，靠牺牲资源环境为代价换取经济增长的老路难以为继，走绿色发展道路是抚州调结构、转方式、实现可持续发展的必然选择。因此必须牢固树立绿色发展理念，大力推进生态文明建设，最大限度地巩固好、发挥好抚州的生态优势，把“绿水青山”变为“金山银山”。

　　二是必须突出改革创新。改革激发活力，创新驱动发展。实践证明，通过全面深化改革,着力推进政府职能、投资、财税、市场等重点领域和关键环节改革,可以释放经济发展活力；通过深入实施创新驱动发展战略，特别是科技创新引领产业向高端层次迈进，可以提升产业竞争力。因此必须突出问题导向，敢闯敢试，在改革创新中破解难题、在创新驱动中开辟新路。

　　三是必须加快推进项目建设。项目建设事关增长和后劲，事关发展和民生，事关全局和长远。实践证明，只有实施项目带动战略，抚州经济才能做大总量、增强后劲，在新一轮发展中掌握稳步前行的主动权。因此必须牢固树立抓项目就是抓发展、抓大项目就是抓大发展的理念，凝聚发展合力，强化责任担当，加快推进项目建设，为科学发展提供坚实支撑。

　　四是必须坚持以人民为中心的工作导向。民之所望，政之所向。实践证明，只有坚持以人民为中心的工作导向，才能真正收获科学发展，才能真正让百姓拥有更多获得感。因此必须始终践行群众路线，强化久久为功意识，多做打基础、利长远的工作，让老百姓过上更有保障、更有尊严的幸福生活。

　　五是必须切实提高政府依法行政水平。政府作为推动经济社会发展的执行者和操作者，必须正确行使人民赋予的权力。实践证明，只有提高政府的依法行政水平,接受人民群众监督,才能真正做到执政为民。因此必须强化法治意识，提升依法行政能力，运用法治思维和法治方式来深化改革、推动发展、化解矛盾、维护稳定。

　　各位代表，发展历程饱含奋斗，发展成果来之不易。这是省委、省政府和市委正确领导的结果，也是全市上下团结奋进、扎实苦干的结果。在此，我代表市人民政府，向全市广大干部群众，向各民主党派、工商联、无党派、新阶层、人民团体、社会各界人士，向中央、省驻抚单位，驻抚部队和武警、消防官兵，向外来投资者和建设者，向所有关心、支持和帮助抚州发展的同志们、朋友们，致以崇高的敬意和衷心的感谢！

　　回顾五年来的工作，我们也清醒地看到，抚州经济社会的发展仍存在着不少困难和问题：经济总量小、人均水平低的状况尚未得到根本改变；投资、出口增幅持续回落，消费增长动力不足；部分经济指标运行质量不高，经济运行面临较大下行压力；实体经济运行困难，生产经营成本上升，部分企业效益下降或亏损；科技创新能力不强，人才总量不足，高层次人才特别是高水平领军人才紧缺；产业结构不合理，结构调整任务艰巨；社保、环保、就业、就学、就医、扶贫、住房保障、食品安全等公共服务与人民群众的期待还有较大差距；一些部门办事效率低下，工作执行力不强，一些干部思想观念、能力素质还不适应发展要求；一些基层干部损害群众利益的问题还时有发生等等。我们必须下定决心，采取得力措施，切实解决好这些矛盾和问题。

　　二、砥砺奋进，谱写科学发展绿色崛起崭新篇章

　　各位代表，前不久闭幕的市第四次党代会作出了“科学发展、绿色崛起，全面建成小康新抚州”的战略部署。市政府将全面落实市委的战略决策。今后五年政府工作的总体要求是：高举中国特色社会主义伟大旗帜，以马列主义、毛泽东思想、邓小平理论、“三个代表”重要思想和科学发展观为指导，深入贯彻习近平总书记系列重要讲话精神，按照“五位一体”总体布局、“四个全面”战略布局的要求，坚持以新发展理念谋划抚州发展，以生态文明建设引领抚州发展，以项目带动为抓手，以科技创新为动力，以作风建设为保障，坚定不移走科学发展、绿色崛起之路，全面建成小康新抚州。

　　主要奋斗目标是：到2020年，全市生产总值达到1800亿元，年均增长9.5%左右；财政总收入达到266亿元、力争突破300亿元，年均增长10%以上；规模以上工业增加值占工业增加值的比重达85%以上；500万元以上固定资产投资完成2100亿元，年均增长14%；社会消费品零售总额达到700亿元，年均增长11%；出口总额达到21亿美元，年均增长5%；实际利用外资达到6.5亿美元，年均增长10%；城镇居民人均可支配收入按当年价格计算，达到40000元，年均增长9.5%；农村居民人均可支配收入按当年价格计算，达到19000元，年均增长10.5%；新增城镇就业20万人，城镇化率达到52%。为了完成上述目标任务，我们将采取以下措施：

　　（一）坚持转型发展，推动产业迈向中高端。

　　围绕加快构建绿色产业体系，全力打好产业升级攻坚战。坚持一手抓科技创新，推动汽车及零配件、生物医药、建筑建材、食品加工、电子信息、有色金属加工产业发展升级；一手抓新经济发展，积极培育壮大文化创意、休闲旅游、中医药、健康养生、现代物流、信息经济等新兴产业，着力打造新的经济增长引擎。

　　1.促进工业转型升级。依靠科技创新，调整产业结构，壮大工业经济。力争到2020年全市规模以上工业主营业务收入突破3000亿元。培育壮大主导产业。抚州高新区、各县（区）要咬住首位产业、重点产业，深入推进主导产业招商，着重引进产业龙头企业、骨干企业和上下游配套企业，不断延长、壮大产业链条，促进产业集群化发展。到2020年，全市六大主导产业主营业务收入突破2600亿元。实施百亿企业培育工程。通过政策聚焦、资源聚焦、服务聚焦，推动博雅欣和、迪比科新能源动力电池、森鸿电子信息、海利不锈钢、自立铜业、精品铜科等一批企业跃上百亿台阶。改造提升传统优势产业。依靠科技创新，推动传统产业转型升级，通过淘汰落后、兼并重组等手段，推动机械制造、建筑建材、纺织服装等传统优势产业全面升级。发展战略性新兴产业。利用大数据、云计算、互联网、物联网等新一代信息技术，推动产业研发、模式、业态等创新变革。进一步培育壮大生物医药、电子信息、新能源新材料等战略性新兴产业。到2020年，实现全市战略性新兴产业规模倍增、高新技术企业倍增、示范基地倍增，战略性新兴产业增加值占工业增加值比重达到30%以上。

　　2.促进农业“接二连三”。推动农业由生产向加工、销售、服务等产前产后延伸，形成一产、二产、三产互动融合发展格局。加快现代农业示范园区建设。重点推动临川国家级现代农业示范园区建设，在建好现有7个省级示范园区基础上，再新增4个省级示范园区，积极创建抚州全市域国家级现代农业示范区。做大做强农业龙头企业。重点围绕南丰蜜桔、广昌白莲、金溪香精香料、南城水产、崇仁麻鸡、东乡生猪、资溪白茶、乐安油茶、黎川香榧等优势产业，培育和壮大一批龙头企业。到2020年，全市规模以上农产品加工企业达到300家，产值突破500亿元。大力培育农业品牌。实施好“生态抚州、绿色产品”品牌战略，加强农产品商标注册和保护，建立全程可追溯质量管理体系，加强农产品基地标准化建设，做大做强农业优势品牌，整合区域公众品牌，让抚州绿色、有机的“金字招牌”更加响亮。

　　3.促进服务业跨越发展。坚持一手抓现有传统服务业提档升级，一手抓现代服务业培育壮大。到2020年，服务业增加值占生产总值的比重达到45%以上。加快旅游强市建设。树立全域旅游理念，按照全产业链要求，大力发展以文化、休闲、度假、养生和体验为主的旅游产品，把旅游业打造成为新经济的重要支柱产业。加快构建“一区一带六极”的旅游发展大格局，努力把抚州打造成为长三角、珠三角、中三角、闽东南沿线城市的休闲旅游度假后花园。加快文昌里历史文化街区、临川温泉景区、大觉山景区、麻姑山景区、浒湾书铺街等一批重点旅游景区建设，五年内，力争创建5A级旅游景区2个以上，4A级旅游景区20个以上，实现县县都有重点景区。大力发展特色民宿经济，推进乡村旅游休闲业提质升级。每个县（区）每年打造100套以上精品民宿。到2020年，全市旅游接待人数突破7000万人次，旅游总收入突破800亿元。加速电子商务发展。促进电子商务与实体经济、百姓生活深度融合。培育电子商务应用示范企业，大力发展农村电子商务，将黎川、广昌建成全国电子商务进农村综合示范县。到2020年，电子商务交易额突破800亿元。大力发展现代金融业。推进金融改革创新。完善金融服务体系，大力引进传统金融、新金融、类金融企业，支持小额贷款及担保公司发展。大力推动金融产品创新，发展公（私）募金融，做大产业引导基金，积极稳妥推进互联网金融产业发展。拓宽企业直接融资渠道，推动更多企业在主板、“新三板”、股权交易中心上市，加快建立多层次资本市场。积极发展健康养老产业。加快国家养老服务业综合改革试点市、全国医养结合试点市建设，推动养老与医疗、体育、教育、旅游等融合发展。市本级抓好市老年公寓项目二期、临川温泉景区、金山寺景区健康养老基地等一批示范项目建设，引导和支持社会力量在各县（区）加快建设健康养老项目。加快发展中医药产业。扶持中药材生产基地建设，重点培育扶持中药骨干企业扩大规模。力争省级中医药产业基地落户抚州。加快中医药人才培养，全面促进中医药治疗技术应用，切实提高中医医疗服务能力，大力发展中医药文化旅游，力争打造中医药千亿级产业。做强文化创意产业。全力创建国家级文化产业示范区，做好文化创意产业发展规划。建立文化创意产业发展引导基金，培育一批特色鲜明的文化创意产业园区。大力发展演艺业，引进文化基础设施综合运营商，争取全国戏剧制作中心落户抚州，打造区域性文化创意产业中心。到2020年，文化产业增加值力争突破200亿元，占GDP的比重达到8%以上。

　　（二）坚持创新发展，增强区域发展竞争力。

　　向科技创新要动力、要活力、要竞争力。到2020年，科技综合实力在全省位次前移2—3位，科技进步贡献率提高到60%以上。

　　1.加快体制机制创新。深化行政审批制度改革。继续加大简政放权力度，推进放管结合、优化服务，努力把抚州打造成江西审批效率最高的设区市之一，推行“互联网+政务”的为民服务新模式。全面推进行政服务和便民服务事项下放到乡（镇）一级。深化财政体制改革。完善县级基本财力保障机制，探索建立符合乡镇财政收入能力评估机制，切实增强对乡镇财力保障能力。深化农业农村改革。健全农村承包土地经营权流转制度，加快覆盖市、县、乡农村产权综合交易平台建设。积极推进农村集体经济组织产权制度改革、农村土地经营权、农村宅基地改革和农民住房财产权抵押担保贷款等试点。深化国资国企改革。推进市属经营性国有资产集中统一监管，进一步做大做强市投资集团、工创投、文旅投、交建投等市属国有投资运营企业。加大力度推广政府和社会资本合作模式，争取PPP项目在基础设施、城镇建设、社会事业、生态环保等领域全面推行。

　　2.打造创新创业平台。鼓励企业开展产学研用合作，增强科技创新能力，全力支持企业与高校或科研院所建立长期合作关系。巩固提升和新建一批重点（工程）实验室、企业技术中心、工程（技术）研究中心等重大创新平台。利用“互联网+”，积极发展众创、众包、众扶、众筹等新模式，大力扶持大学生创业基地等创业载体发展，打造一批新兴产业“双创”示范基地。

　　3.发挥各类人才作用。深入推进“双返双创”活动，大力实施项目回迁、人才回乡和资金回流“三大工程”，到2018年，全面完成“十百千万”的工作目标。加大政策扶持力度，实施创新创业高层次人才引进计划，推行柔性引进等方式，根据全市重点产业发展方向，每年引进一批高层次创新创业人才。加快现代职业教育体系建设，支持骨干企业与高校开展人才联合培养，推动一批高校向应用技术型高校转型。

　　（三）坚持协调发展，构建统筹协同新格局。

　　发挥比较优势，着力补齐短板，培育发展后劲，持续增强发展的整体性、协调性。

　　1.统筹重大项目建设。围绕打好基础设施提升攻坚战，谋划实施一批打基础、管长远、补短板、增后劲、惠民生的重大项目。推进一批交通基础设施项目建设。加快鹰梅铁路、吉抚武铁路、南昌至抚州城际铁路、南丰至瑞金城际铁路项目前期工作，力争早日开工，推进向莆铁路全线开通货运班列。加快东外环高速建设，积极开展丰城—崇仁—宜黄—南城—南丰对接海西高速、抚州西外环高速、贵溪至资溪高速、新余至乐安至广昌高速前期规划设计工作，形成“三纵三横一联”高速公路网。启动抚州至金溪、崇仁、南城快速通道的规划建设。加快推进抚州机场前期工作，争取“十三五”前期开工建设，推动南丰、资溪、乐安、南城等县通用机场前期工作。加快市中心城区BRT建设，开通抚州至崇仁城际公交。启动恢复抚河分段通航，把抚河打造成水上旅游黄金水道。推进一批能源基础设施项目建设。积极争取抚州大唐电厂二期工程开工建设，建成500千伏“一纵一横”骨干网架，实现220千伏电网县（区）全覆盖。加快推进新一轮农村电网改造升级。加快天然气管网建设，扩大长输管网覆盖面，争取实现县县通管道天然气。推进一批农村基础设施项目建设。实施农村公路畅通安全工程，建设25户以上自然村通村组公路。推进廖坊水利枢纽灌区二期工程、东乡井山水库、黎川长滩水库及抚河、宜黄河重点河段治理工程等重点水利基础设施建设。大力实施农村饮水提质工程。

　　2.统筹城乡一体化发展。大力推进以人为核心的新型城镇化，全面提升城镇化发展质量。加快推进新型城镇化。坚持规划先行，推动全域空间“多规合一”。争取尽快完成东乡撤县设区，并进一步拓展成果，全力推进已获批的国家新型城镇化综合试点工作，做大做强以抚州高新区、临川、东乡、金溪、崇仁为主体的核心增长极。实施东扩南拓西联北控战略，推动上顿渡片区、文昌里片区、东乡城区与市中心城区融合发展。扎实推进“智慧城市”建设，加快建成“全光网城市”，积极申报“海绵城市”试点。全力创建国家卫生城市、国家生态园林城市、国家历史文化名城。推动县域经济发展升级。实施科学发展分类考核，促进各县（区）因地制宜发展特色经济，做大总量、壮大财源，力争县县财政收入超10亿元，争取一半以上的县过20亿元。积极推进省直管县改革试点，扩大县级经济社会管理权限。加快秀美乡村建设。全面推进新一轮乡镇总体规划、控制性规划修编和修建性规划编制工作，完成中心村和30户以上自然村的村庄规划编制工作。严格执行规划，加强村镇建设管理，加快实现城乡一体化。持续开展农村环境综合整治行动，努力创建秀美、和谐、宜居的农村环境。加快特色小镇建设，到2020年，建成特色小镇23个，其中各县（区）至少打造2个，抚州高新区至少打造1个。每年安排新农村建设村点1000个左右，5年完成300个左右中心村整治建设。

　　3.统筹物质文明和精神文明发展。坚持用中国梦和社会主义核心价值观凝聚共识、汇聚力量。推进群众性精神文明创建。扎实开展“做文明抚州人”市民文明大行动，全面推进文明城市、文明村镇、文明单位、文明家庭等创建活动。加强农村精神文明建设，利用优秀的传统文化和乡规民约，加强农村道德教育,培育文明新风,提高农民的道德水平和科学文化素质。推动解决农村环境“脏乱差”，严厉整治农村封建迷信、诚信缺失、铺张浪费、大操大办、厚葬薄养、盲目攀比等突出问题。丰富群众精神文化生活。完善公共文化服务体系，加大公共文化产品供给，更多向社会力量购买公共文化服务。加快启动市级全媒体中心（市传媒中心）建设，加快中国戏曲博物馆、科技馆、青少年活动中心、市级档案新馆等工程建设。提升临川文化影响力。深入挖掘临川文化的深刻内涵，增强文化自信和文化自觉。做大做强汤显祖文化品牌，加快汤显祖作品进教材和多语种翻译工作落地，办好中国（抚州）汤显祖艺术节、戏剧论坛等系列文化活动，繁荣地方戏剧文化发展，打造中国戏曲之都。谋划举办好2019年曾巩诞辰1000周年纪念活动和2021年王安石诞辰1000周年活动。重点加强非物质文化遗产的保护、开发和利用，积极申报国家和省级历史文化名城（街区）、名镇、名村和中国传统古村落。

　　（四）坚持开放发展，提高经济发展外向度。

　　加快实施“融入南昌、对接海西、建设向莆经济带”发展战略，积极融入“一带一路”和长江经济带国家战略，打造开放型经济升级版。

　　1.深化昌抚合作。推动基础设施对接。进一步完善昌抚一体化的交通网络。加快推进昌抚城际铁路规划，力争2017年开工建设，“十三五”期间建成通车。推动产业合作对接。以昌抚合作示范区建设为平台，打造与南昌合作的战略支点，在文化教育、休闲旅游、宗教文化、养生养老、城镇开发上实现差异化错位发展。推动公共服务对接。在实现昌抚通信、金融、公积金同城化的基础上，主动加强与南昌在公共服务领域的对接，加快全面实现医疗、社保、户籍等同城化。推动生产要素对接。继续破除要素流通中的各种行政壁垒，改善市场环境，降低交易成本，加快形成统一开放、公开透明、竞争有序的市场秩序和政策体系。

　　2.推进对接海西。努力构建江西出海便捷战略大通道，将抚州建成江西对接“21世纪海上丝绸之路”的桥头堡。实现交通更加便利。全面参与布局和协同建设跨省铁路、高速公路、港口码头、机场和物流园区等重大基础设施项目，形成立体交通网络，构建通江达海大通道。争取福建等沿海地区在抚州设立“无水港”，与湄洲湾港、福州港等共建专属港区。实现通关更加便捷。加快江西出入境检验检疫局抚州办事处检测中心、金溪香精香料检测实验室等项目建设，规划建设南丰公路口岸作业区，加快设立抚州海关步伐，适时建设保税物流中心。完善公铁联运、海铁联运、陆海联运等多式联运集成设施。实现产业更加互补。推进赣闽台产业合作示范区建设，主动对接闽台等地临港产业。引导生产要素向向莆铁路沿线区域集聚，形成一批外向型产业集群和产业基地。

　　3.加大招商力度。着力提升招商引资精准度和成功率。顺应大趋势。把握好浙闽粤等沿海发达地区产业转移的大趋势，实行“点对点”、“敲门式”招商。大力引进台闽出口加工贸易、电子信息、物流服务和农产品加工企业。引进大企业。深入推进“民企入抚、央企入抚、外企入抚”和“双返双创”活动，五年力争引进世界500强企业5家，央企20家，国内民营500强企业30家。在引进顶天立地大企业的同时，采取政策支持措施，引进做大铺天盖地的中小企业。配套大产业。聚焦六大主导优势产业及六大新兴产业，围绕产业发展“鱼骨图”，开展产业配套招商，延伸产业链、提升价值链。推动各县（区）打造特色鲜明、具有品牌效应的“一县一业”，到2020年，力争抚州高新区主营业务收入过千亿元，工业发展重点县（区）工业主营业务收入过500亿元。

　　4.扩大对外开放。鼓励我市企业和个人对外进行投资，推动比较优势明显的企业到境外投资办厂，建立海外生产基地和产业园区。组织和引导本地建筑企业“走出去”，积极开展对外承包工程，打响临川“建筑之乡”品牌。

　　（五）坚持绿色发展，打造生态文明样板区。

　　以抚河流域生态保护及综合治理项目为主线，推进生态文明建设体制机制创新，加快打造美丽江西“抚州样板”。

　　1.打造生态环境治理“抚州样板”。全面实施抚河流域生态保护及综合治理项目，力争打造成为全省、全国的生态治理榜样工程。深入实施全市域封山育林，争取新增崇仁、乐安、金溪纳入国家重点生态功能区。按照“生态文明+特色小镇+绿色生活方式”的标准，着力抓好抚河干流沿岸6个县（区）的36个生态示范村镇建设。深入开展“净空、净水、净土”行动，确保空气质量优良天数占比位居全省前列。争创国家级自然保护区2个、国家级森林公园3个、国家级湿地公园4个以上。

　　2.打造绿色生产生活方式“抚州样板”。发展低碳经济。推动产业绿色低碳循环发展，引导、支持企业节能减排、清洁生产，大力发展节能环保产业。同时，严把项目落地环境关，制订出台市、县招商引资“负面清单”，严禁污染项目落户。推动工业园区循环经济试点。发展生态循环农业，促进农业可持续发展。推进商业、物流、餐饮住宿、旅游等行业低碳节能。完善节约用地考核评价机制，突出提高工业用地投资强度，提高土地使用效率。推广绿色生活。积极开展生态市、生态县、生态乡镇创建活动，建设一批绿色机关、绿色学校、绿色社区、绿色企业和绿色家庭。推行政府绿色采购，倡导绿色出行、绿色消费，加快建设资源节约、环境友好型社会。

　　3.打造完善绿色制度“抚州样板”。构建生态文明绩效评价体系。按照各地主体功能定位，建立健全对各县（区）有区别、有侧重的绩效评价考核体系。创新生态环境保护责任机制，全面推行“河长制”、“山长制”、“路长制”，构建市、县、乡、村、组五级责任体系。加快编制自然资源资产负债表，建立健全领导干部任期生态文明责任制度、自然资源资产和环境责任离任审计及生态环境损害责任终身追究制。建立多元化生态补偿机制。开展抚河源头生态补偿试点，推动建立跨区域的横向生态补偿制度和流域内的纵向生态补偿制度。健全生态环境保护市场化机制。完善土地、矿产、森林、湿地等资源有偿使用制度。建设生态云平台，探索开展林业碳汇交易试点和抚河流域排污权交易试点，形成有利于资源节约和环境保护的利益导向机制。

　　（六）坚持共享发展，增强人民群众获得感。

　　坚持以人民为中心的工作导向，以保障和改善民生为重点，以提高人民幸福指数为目标，构建公平公正、共建共享的社会发展新机制。

　　1.实施精准脱贫攻坚工程。全力打好脱贫攻坚战，做到真扶贫、扶真贫、真脱贫。到2018年底，全面完成全市2个贫困县、223个贫困村和12.6万贫困人口脱贫摘帽的目标。实施产业扶贫。强化农民合作社、龙头企业与贫困户利益连结机制，支持贫困村“一村一品”扶贫工程，力争全市所有贫困村形成“一村一个特色产业”格局，覆盖80%以上贫困人口。实施保障扶贫。完善农村最低生活保障制度，对无法依靠产业扶持和就业帮助脱贫的贫困群众实行政策性保障兜底，全部纳入最低生活保障范围。进一步加大对特困人群的医疗费用救助力度，提高大病保险水平，降低贫困群众医疗负担。实施安居扶贫。对生产生活条件恶劣地区实施扶贫移民搬迁工程。将贫困户全部纳入农村住房保障体系。

　　2.实施民生实事建设工程。促进就业创业。加强对灵活就业、新就业形态的支持，促进劳动者自主就业；稳定并扩大就业规模，城镇登记失业率控制在5%以内。完善社会保障。整合城乡居民医疗保险制度，探索建立城乡居民大病保险制度，不断提高大病医疗保障水平。健全最低生活保障、特困人员供养等社会救助制度，推进重大疾病医疗救助、困难群众殡葬救助、困难家庭救助等专项救助制度建设。健全住房保障体系。推进城市棚户区、危旧房、城中村改造，鼓励货币化安置，实施以购代建。健全保障性住房投资运营管理机制，完善准入和退出管理机制。扩大住房公积金支付范围，增强住房保障能力。

　　3.实施社会事业发展工程。以促进教育持续协调发展为目标，加快临川教育集团转型升级，加快临川一中、临川二中、抚州一中三所实验学校建设步伐。全面实施义务教育免试就近入学，实现县域义务教育基本均衡。组建抚州职业教育联盟，提升职业教育水平。加快高等教育事业发展，支持东华理工大学、南昌大学抚州医学院等驻抚高校提升发展，积极推动江西中医药高专“专升本”和崇仁师范学校升格为抚州幼儿师范高等专科学校。推行乡镇卫生院标准化建设。全面深化公立医院综合改革。完善市中心城区医疗服务体系，加快推进南昌大学抚州医学院附属医院、市妇女儿童医院、第一人民医院老院区独立建院（三级医院）、临川区第一人民医院改扩建升级三级医院、市中医院、市康复医院、“120”急救中心等项目建设。

　　4.实施公共安全保障工程。深入推进“平安抚州”建设，健全重大决策社会稳定风险评估机制。完善社会矛盾排查预警和多元化解决机制。加快推进创新引领，加强社会治安综合治理，依法严厉打击各类违法犯罪活动，提升公众安全感。强化食品药品监管，健全最严格的食品药品监管制度和体系。严格落实“党政同责、一岗双责、失职追责”的安全生产责任制度。健全监测预报、灾害防御、应急救援体系建设，提高灾害应对处置能力。

　　与此同时，加强国防动员和后备力量建设，推进军民融合深度发展，持续开展“双拥”模范城（县）创建工作，巩固和发展军政军民团结。

　　三、恪尽职守，全面加强政府自身建设

　　面对艰巨而繁重的改革发展任务，面对人民群众对美好生活的热切期盼，我们必须全面加强政府自身建设，切实担当起“科学发展、绿色崛起，全面建成小康新抚州”的历史责任。

　　（一）坚定信念，恪守政治立场。牢固树立政治意识、大局意识、核心意识、看齐意识，始终在思想上政治上行动上与以习近平同志为核心的党中央保持高度一致。加强政治理论学习，完善市政府党组学习制度，把学习贯彻习近平总书记系列重要讲话精神作为首要政治任务，自觉用重要讲话精神武装头脑、指导实践、推动工作。加强市政府党组自身建设，严格遵守党内政治生活准则,带头参加“三会一课”，自觉执行领导干部双重组织生活规定。真正把严守政治纪律和政治规矩作为政治上的生命线，以更强的党性意识、政治自觉和组织观念，扎实做好政府各项工作。

　　（二）敢于担当，恪守为民宗旨。坚决克服“因循守旧、不思进取，不求有功、但求无过”的不良倾向，积极倡导知难而进、迎难而上、破难前行的优良作风，遇到矛盾不绕、面对困难不躲、解决问题不拖，对改革发展中遇到的困难问题敢亮剑、能攻坚、打胜仗。全面整治“慵、懒、散、拖”，加大履职尽责监察力度，坚决纠正和惩处不作为、慢作为、乱作为。狠抓政府执行力建设，严格绩效管理，强化责任落实。

　　（三）依法行政，恪守法治精神。扎实推进“七五”普法，坚持运用法治思维和法治方式深化改革、推动发展、化解矛盾。严格依照法定权限和程序行使权力、履行职责。加强政府立法，完善依法行政制度体系。推进行政决策科学化、民主化、法治化。积极推行政府法律顾问和公职律师制度。自觉接受市人大及其常委会的依法监督、市政协的民主监督和社会各方面监督，认真办理人大代表建议、议案和政协提案。深入实施“阳光政务”，全面推进政务公开。

　　（四）廉洁从政，恪守纪律红线。坚持把纪律和规矩挺在前面，全面落实政府系统党风廉政建设主体责任制，带头落实“一岗双责”。严格落实中央“八项规定”、国务院“约法三章”。加大监察、审计、财政监督力度，强化对重大工程、重点行业、重要环节和重大资金的监管，严肃查处各类违纪违法案件，抓好企业和群众身边的“微腐败”整治，着力营造廉洁高效的政务环境。

　　各位代表！站在新起点，面对新使命，蓝图已绘就，奋进正当时。让我们紧密团结在以习近平同志为核心的党中央周围，在省委、省政府和市委的坚强领导下，解放思想，锐意进取，团结拼搏，扎实苦干，为实现“科学发展、绿色崛起，全面建成小康新抚州”而努力奋斗！
